# Critical Mutation Rate has an Exponential Dependence on Population Size for Eukaryotic-length Genomes with Crossover

**DOI:** 10.1038/s41598-017-14628-x

**Published:** 2017-11-14

**Authors:** Elizabeth Aston, Alastair Channon, Roman V. Belavkin, Danna R. Gifford, Rok Krašovec, Christopher G. Knight

**Affiliations:** 1 School of Computing and Mathematics, Keele University, Keele, Staffordshire, UK; 2 School of Engineering and Information Sciences, Middlesex University, London, UK; 3 Faculty of Science and Engineering, The University of Manchester, Manchester, UK

## Abstract

The critical mutation rate (CMR) determines the shift between survival-of-the-fittest and survival of individuals with greater mutational robustness (“flattest”). We identify an inverse relationship between CMR and sequence length in an *in silico* system with a two-peak fitness landscape; CMR decreases to no more than five orders of magnitude above estimates of eukaryotic per base mutation rate. We confirm the CMR reduces exponentially at low population sizes, irrespective of peak radius and distance, and increases with the number of genetic crossovers. We also identify an inverse relationship between CMR and the number of genes, confirming that, for a similar number of genes to that for the plant *Arabidopsis thaliana* (25,000), the CMR is close to its known wild-type mutation rate; mutation rates for additional organisms were also found to be within one order of magnitude of the CMR. This is the first time such a simulation model has been assigned input and produced output within range for a given biological organism. The decrease in CMR with population size previously observed is maintained; there is potential for the model to influence understanding of populations undergoing bottleneck, stress, and conservation strategy for populations near extinction.

## Introduction

**Figure 1:**
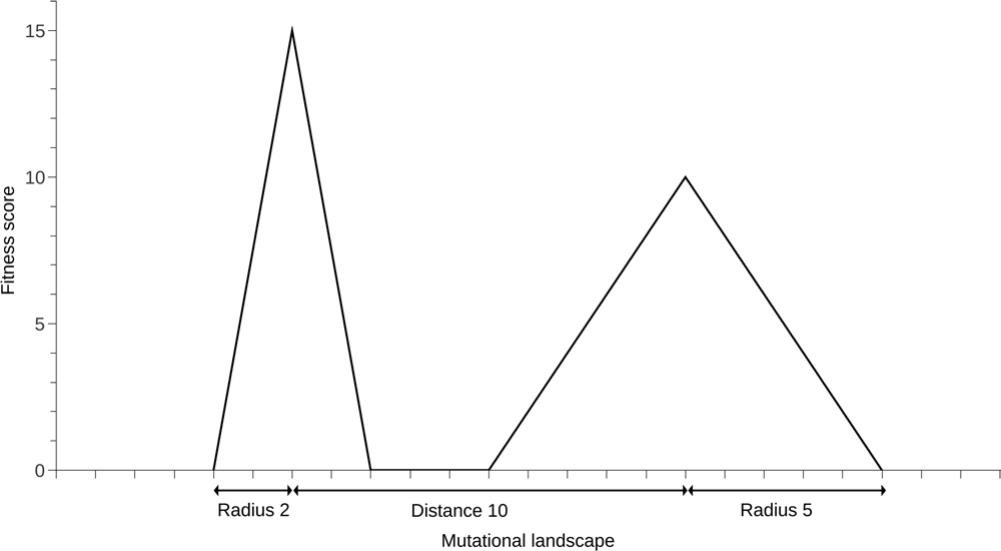
Two-peak fitness landscape with one narrow peak of high fitness (peak 0), and one broader peak of lower fitness (peak 1). Each step on the x axis represents a single base mutation. Diagram adapted from Wilke^4^.

Fitter genotypes can be outcompeted by genotypes with greater robustness when the mutation rate exceeds a critical mutation rate (CMR); in terms of fitness landscapes, narrow high fitness peaks may be lost, while broader, lower peaks are maintained by a population of reproducing sequences. The greater the robustness, the smaller the effect of a mutation on fitness^1^. Most non-neutral mutations are detrimental to fitness^2^, therefore robustness can limit the damage each time a mutation occurs. This so called “ survival-of-the-flattest” has been observed in *in silico* evolving systems^3–5^, in theory^5^, in simulated RNA evolution^6^, and in RNA viruses^7^. CMR reduces exponentially at low population sizes in both haploid^8^ and diploid populations^9^, where the CMR is defined as the mutation rate at which 95% of runs lead to all individuals in the population losing the fitter, narrower peak in a two-peak landscape within 10,000 generations (Fig. 1). As population size falls, the CMR above which fitter alleles are lost transitions unexpectedly from near-constant to drop exponentially for small populations; the previous assumption in evolutionary biology was that the CMR remained near-constant irrespective of population size^10^. The relationship between population size and CMR closely reproduces the established mathematical relationship between population size and the mutation rate above which individuals lose all of the peaks, the “error threshold”^9^. The observation that CMR decreases with population size suggests small populations may be subject to survival-of-the-flattest at mutation rates lower than previously assumed. Endangered species often consist of small and fragmented populations; fragmentation, habitat destruction, and environmental stresses such as pollution, all contribute to a reduction in population size, which in turn has major effects on population genetics and demography^11–13^. Small population effects are relevant across the range of organisms, even microorganisms, that typically occur at large population sizes. Microbes can experience populations just as small as macro-organisms, for instance at the point of infection of a new host, or close to removal by a host immune system, and that will affect their evolution^14^. However, biological organisms typically have lengths and numbers of genes orders of magnitude higher than those used in models of error thresholds or CMRs, so how relevant such models are to real biological populations remains an open question. To bridge the gap between artificial and biological evolution it is paramount that a model can be assigned parameter values that respect biological reality. Here we extend CMR theory to consider relevance of the parameters of gene length and the number of genes in organisms capable of undergoing chromosomal crossover, while respecting biologically-relevant mutation rates for eukaryotic organisms (Table 1).

**Table 1:** Mutation rates for various eukaryotic species.

Species	Genome size (Mbp)	Mutation rate	Base/genome	Unit	Source
Human	3080	$1\times {10}^{-8}$–$2.5\times {10}^{-8}$	Per base	Per generation	49,56,57
Human	3080	$1.75\times {10}^{2}$	Per genome	Per generation	49
Human	3080	$5\times {10}^{-11}$–$6\times {10}^{-2}$	Per base	Per cell division	46,57
Human	3080	$1.6\times {10}^{-1}$	Per genome	Per cell division	46
Human (Y chromosome)	58	$3\times {10}^{-8}$	Per base	Per generation	61
Human, chimpanzee	3080	3	Per genome	Per generation	50
$D.$ *melanogaster*	120	$4.65\times {10}^{-9}$–$6.2\times {10}^{-8}$	Per base	Per generation	47,48,51,57
$D.$ *melanogaster*	120	$9.9\times {10}^{-1}$–1.2	Per genome	Per generation	47,50
$Drosophila$ spp.	120	$7\times {10}^{-2}$	Per genome	Per generation	50
$D.$ *melanogaster*	120	$1.3\times {10}^{-10}$–$3.4\times {10}^{-10}$	Per base	Per cell division	46,57
Quail, chicken	1050	$4.9\times {10}^{-1}$	Per genome	Per generation	50
Sheep, cow	2870	$9\times {10}^{-1}$	Per genome	Per generation	50
Old World Monkey		1.9	Per genome	Per generation	50
Mouse, rat	2640	$9.1\times {10}^{-1}$	Per genome	Per generation	50
Mouse	2640	$1.8\times {10}^{-10}$	Per base	Per cell division	46
Mouse	2640	$1.1\times {10}^{-8}$	Per base	Per generation	46
$S.$ *cerevisiae*	12.1	$3.3\times {10}^{-10}$	Per base	Per generation	57
$S.$ *cerevisiae*	12.1	$3.3\times {10}^{-10}$	Per base	Per cell division	52
Average mammalian		$2.2\times {10}^{-9}$	Per base	Per genome/year	55
Mammalian upper bound		$2.61\times {10}^{-9}$	Per base	Per genome/year	55
$C.$ *elegans*	100	$8.4\times {10}^{-9}$–$2.1\times {10}^{-8}$	Per base	Per generation	47,53,58
$C.$ *elegans*	100	2.9	Per genome	Per generation	52
$A.$ *thaliana*	157	$7.1\times {10}^{-9}$	Per base	Per generation	54
$A.$ *thaliana*	157	$6.5\times {10}^{-9}$	Per base	Per generation	58

Mutation rate estimates were obtained by comparing pseudogenes (genes that do not code for proteins or are never expressed) in humans and chimpanzees^49^, combining the results of theoretical and empirical studies^55^, mutation accumulation and radiation experiments^51^, direct sequencing of the human Y chromosome^61^, computational analysis of genes from species of placental mammals^55^, whole-genome shotgun sequencing of mutation accumulation lines of the fruit fly *Drosophila melanogaster* ^48,51^ and the nematode worm *Caenorhabditis elegans* ^53^, examination of sequence variation in the human genome^56^, scanning the mitochondrial genome of *D. melanogaster* ^47^, study of the complete genome of five *Arabidopsis thaliana* lines^54^, and complete genome sequencing of the yeast *Sacccharomyces cerevisiae* ^52^. Other sources listed contain a range of estimates^57,58^.

Eigen and Schuster^15^ theoretically determined the error threshold in terms of selection pressure and sequence length. Using this model, it was found that longer sequence lengths lead to lower error thresholds in genetic algorithms^16,17^. Nowak^18^ theoretically determined the error threshold in terms of the relative fitness of mutant and wild type, concluding that increasing sequence length will decrease the error threshold. According to the drift-barrier hypothesis^19^, the strength of selection that reduces mutation rate through mutation-selection balance is countered by *Ne*-dependent genetic drift (where *Ne* is effective population size)^20^. Following this population size dependence, we hypothesise that, for increasing sequence lengths, the CMR will decrease with population size, and that this will occur in line with the exponential model identified in Aston *et al*.^9^. Increasing the length of the sequences, and therefore the size of the mutational landscape, will decrease the proportion of the landscape taken up by the peaks (Fig. 1). Assuming peak radii are kept constant, increasing the Hamming distance between the peaks will increase the number of neutral mutations it will take to move from one peak to another. However, the radius of the peaks and the Hamming distance between them are not expected to be independent. While increasing the distance between the peaks will increase the neutral space between them, increasing the radius of the peaks will simultaneously reduce the space. If the distance between the peaks is increased, and the radius of the peaks is also increased by the same magnitude (i.e., scaled by parameter S), there are three potential effects: the CMR will increase as S increases if the reduction in neutral space due to increased peak radius exceeds the increase in neutral space due to peak distance, it will decrease if the opposite is true, or it will stay the same if there is a balance between the two. In terms of the landscape defined in Fig. 1, the combined radii of the peaks is 7, while the neutral space between them is 3. It is therefore expected that the effect of increasing peak radius will outweigh the effect of increasing peak distance; CMR is expected to increase as S increases.

In addition to mutation, individuals can move around the fitness landscape by way of recombination events that involve a reciprocal exchange of genetic material (known as crossover). Recombination has been seen to lower the mutation rate at which the error threshold occurs in viruses^21^. Similarly, Ochoa *et al*. observed that recombination can push the population in a genetic algorithm over the error threshold when the mutation rate is high (such as when it is close to the CMR)^22^. We therefore hypothesise that increasing the number of crossover events that occur during reproduction will increase the magnitude of the CMR. Similarly, increasing the number of chromosomes the genome is split into is also expected to affect the magnitude of the CMR; in the model there is a crossover event per chromosome therefore an increase in chromosome number will result in an increase in the number of crossovers per reproduction. We hypothesise that increasing the number of genes in the simulation (while keeping gene length constant) will lower the CMR as it will increase the overall sequence length. Gene numbers within biological ranges (i.e., 25,000 genes to model *A. thaliana*) are expected to lead to CMRs close to the range of wild-type mutation rates; it is expected biological organisms will be evolving close to the mutation rate that results in the fastest rate of adaptation.

### Key Definitions

#### Critical mutation rate (CMR)

The mutation rate at which 95% of runs lead to all individuals in the population losing the fitter, narrower peak in a two-peak landscape within 10,000 generations. It is the mutation rate at which there is a transition from survival-of-the-fittest to survival-of-the-flattest.

#### Crossover

Recombination event that involves a reciprocal exchange of genetic material.

#### Effective population size

The number of individuals in a population that contribute offspring to the next generation.

#### Error threshold

The mutation rate above which the population loses all of the peaks in the landscape (known as error catastrophe).

#### Fitness score

The value assigned to an individual according to their position in the fitness landscape.

#### Hamming distance

The number of bases different between two sequences.

#### Neutral mutation

A mutation that has no effect on fitness.

#### Peak radius

The Hamming distance between the top of the peak and the point of zero fitness. The greater the radius, the broader the peak.

#### Robustness

The average effect of a specific type of perturbation (such as a *de novo* mutation) on the fitness of a specific genotype. The smaller the change in fitness, the more robust the genotype is to mutation.

#### Survival-of-the-flattest

When individuals with greater robustness to mutation are favoured over individuals with greater fitness.

## Results

The exponential relationship between population size and CMR previously reported^9^ is observed when parameters for the simulation model are set to values within biologically-relevant ranges. The CMR decreased with population size for a range of sequence lengths, peak radii and distance between peaks, crossover events per reproduction, chromosome numbers (with a crossover event per chromosome), and the total number of genes an individual is made up of. The magnitude of the CMR was influenced most by the length of the sequence and the number of genes. When the length and the number of genes was set to 2,000 bp and 25,000 respectively, which are values relevant to the plant *A. thaliana*, the CMR was recorded to be within one order of magnitude higher than the wild-type mutation rate listed for *A. thaliana* in Table 1.

### Increasing the sequence length leads to a decrease in CMR

**Figure 2:**
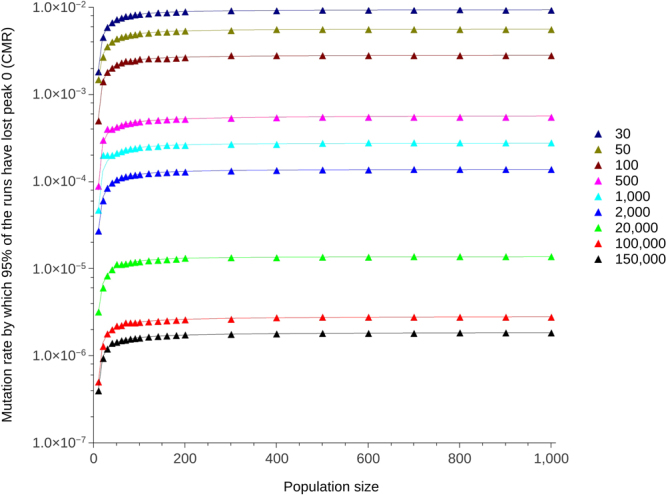
CMR when the simulation model was run for one gene with sequence lengths of 30 up to 150,000 bp (shown in the legend) for population sizes 10 up to 1,000. Peak 0 had a radius of 2 and peak 1 a radius of 5. The Hamming distance between the peaks was 10. The exponential lines were obtained by curve-fitting using R with a least squares method. Error bars are not plotted, but are small, for instance $9.37\times {10}^{-03}$ ($9.317\times {10}^{-03}$–$9.423\times {10}^{-03}$, 95% confidence interval) and $1.84\times {10}^{-06}$ ($1.834\times {10}^{-06}$–$1.846\times {10}^{-06}$, 95% confidence interval) for uppermost (30 bp) and lowest (150,000 bp) points (population size 900) respectively.

Figure 2 shows the CMR for sequence lengths of 30 up to 150,000 bp; increasing sequence length decreases the CMR in a single-gene-per-individual diploid *in silico* evolving system. Increasing the sequence length from 30 up to 150,000 bp lead to the CMR falling by close to four orders of magnitude. Peak 0 and peak 1 were given an initial radius of 2 and 5 respectively, while the distance between the top of the peaks was set to 10 (as per Aston *et al*.^9^). The exponential relationship between CMR and population size presented previously is maintained irrespective of sequence length. Two-way ANOVA indicates that, for increasing sequence lengths, the CMR will decrease with population size in line with the exponential model identified in Aston *et al*.^9^ (p < 0.0001, α = 0.05, F(8,176) = 453.09 for the null hypothesis that there will be no decrease in CMR with population size for increasing sequence lengths)(see Supplementary Table 1). It should be noted that results relating to the relationship between population size and sequence lengths 2,000 and 20,000 (shown as part of Fig. 2) were presented at the Artificial Life 2016 conference^23^.

### Scaling the radius of the peaks and the Hamming distance between them affects the magnitude of the CMR

**Figure 3:**
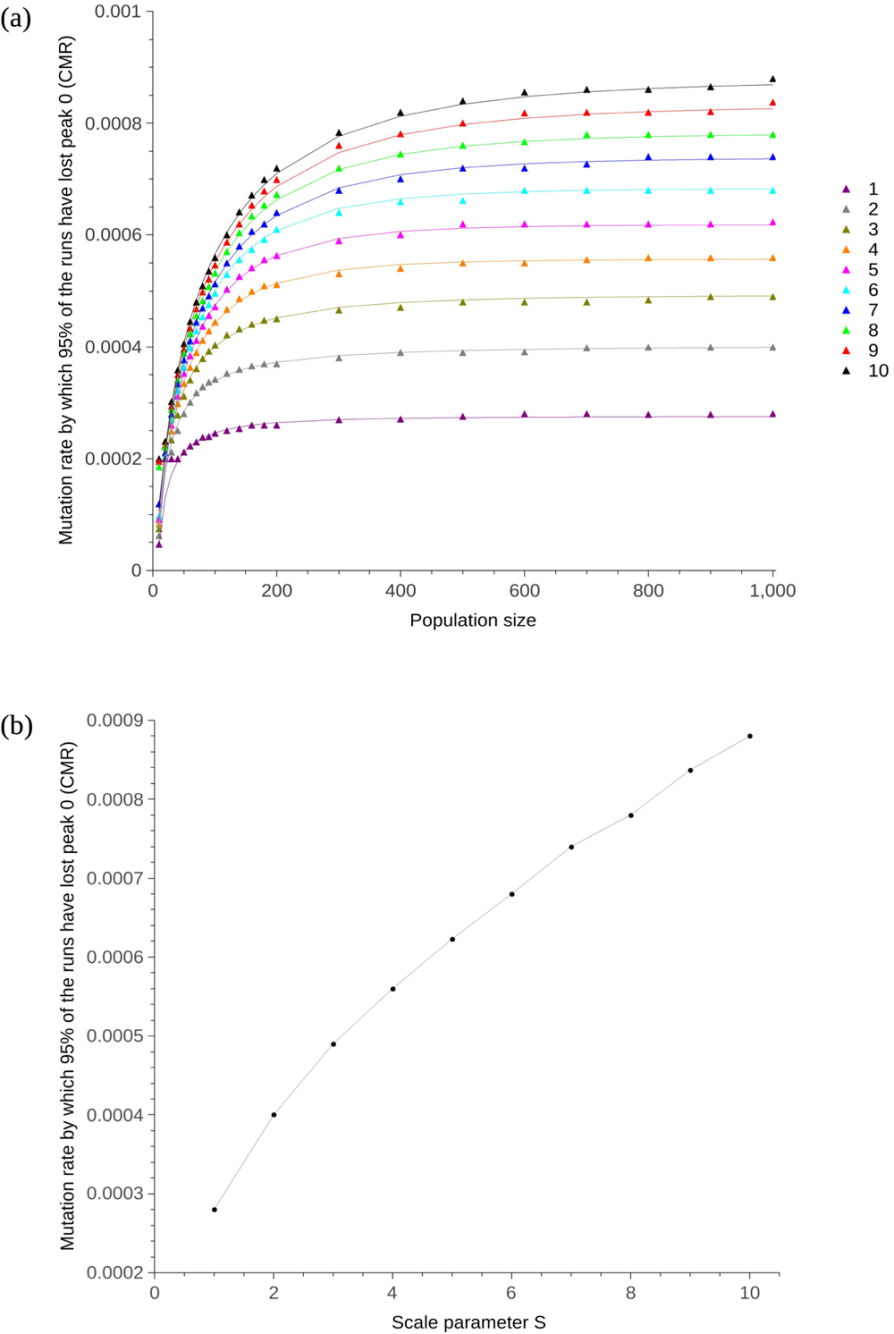
CMR plotted against population size for varying values of scale parameter S. Population size was varied from 10 up to 1,000 and each individual consisted of 1 gene of 1,000 bp in length. Peak 0 and 1 were given a radius of 2 and 5 respectively, while the distance between their peaks was set to 10. (**a**) CMR plotted for population sizes 10 up to 1,000. The radius of the peaks was initially set to 2 and 5 for peak 0 and 1 respectively, with the distance between the top of the peaks set to 10. These values were then scaled by parameter S (shown in the legend). The exponential lines were obtained by curve-fitting using R with a least squares method. Error bars are not plotted, but are small, for instance $8.66\times {10}^{-04}$ ($8.621\times {10}^{-04}$–$8.699\times {10}^{-04}$, 95% confidence interval) and $2.75\times {10}^{-04}$ ($2.740\times {10}^{-04}$–$2.760\times {10}^{-04}$, 95% confidence interval) for uppermost (S = 10) and lowest (S = 1) points (population size 900) respectively. (**b**) CMR plotted for varying values of S when population size is 1,000.

Figure 3 shows that when the radius of peak 0, peak 1, and the distance between them, is scaled by parameter S, increasing S leads to an increase in the CMR. Peak 0 and 1 were given an initial radius of 2 and 5 respectively, while the distance between their peaks was set to 10 (as per previous experiments). Parameter S was increased from 1 to 10 (making the minimum distance 10 and the maximum distance 100; this covers the range given in Table 2). The CMR curves flatten out initially at 0.00028, increasing to 0.00087 when S reaches 10. The exponential relationship between population size and CMR was maintained irrespective of peak width and distance. Two-way ANOVA indicates that the CMR will increase as S increases (p < 0.0001, *α* = 0.05, F(9,198) = 89.07 for the null hypothesis that there will be no increase in CMR with S) (see Supplementary Table 2). Keeping the radius of peak 0 constant at 2, while increasing the radius of peak 1 from 4 up to 5, then up to 6 (not allowing the peaks to meet or overlap), produced results with no statistical significance (p = 0.12, *α* = 0.05, F(2,44) = 2.19).

**Table 2:** Genetic distances between alleles for various genes.

Distance between alleles	Unit	Gene	Source
2.02	% difference	Adh1-1F and Adh1-1S alleles in maize	59
1	Amino acid	Rice blast resistance (R) gene Pi-ta	60
13	Single nucleotide polymorphisms (SNPs)	Pikh allele for rice varieties	62
7			
7			
19			
45			
48			
54			
19			
56			
47			
19	Base pairs	Adh1 alleles of wild barley	63
0, 1, 1	Synonymous, non-synonymous, non-coding SNPs	HMGCR	35
3, 2, 1		HSD3B1	
1, 0, 0		HTR1EL	
2, 3, 9		HTR2A	
0, 1, 0		HTR2C	
2, 0, 0		HTR5A	
1, 0, 0		HTR6	
0, 0, 0		HTR7	
0, 0, 8		IGF1	
0, 0, 1		IGF2	
4, 3, 0		ITGA2B	
4, 3, 0		ITGB3	
0, 1, 2		KLK2	
3, 0, 0		LCAT	
7, 3, 0		LDLR	
4, 3, 4		LIPC	
1, 1, 0		LPL	
1, 0, 0		MAOA	
1, 0, 0		MAOB	
1, 2, 1		MPL	
1, 1, 5		NGFB	
1, 0, 0		NT3	
5, 2, 0		NTRK1	
2, 0, 4		PACE	
1, 2, 1		PAI1	
5, 4, 5		PAI2	
1, 3, 1		PC1	
5, 5, 4		PCI	
0, 0, 0		POMC	
1, 1, 1		PRL	
3, 0, 0		PROC	
1, 0, 0		PROS1	
0, 2, 0		PTAFR	
1, 0, 2		PTH	
0, 0, 13		PTHLH	
5, 8, 0		SELP	
1, 3, 1		SHBG	

The data from Cargill *et al*.^35^ represents polymorphisms in alleles for a subset of human genes (cross-section displayed below). Where there is more than one distance listed per source, the Unit and Source columns are left blank. Where there is multiple data for one gene, the Gene column is also left blank.

### Increasing the number of crossovers per reproduction or the number of chromosomes per genome leads to an increase in CMR

**Figure 4:**
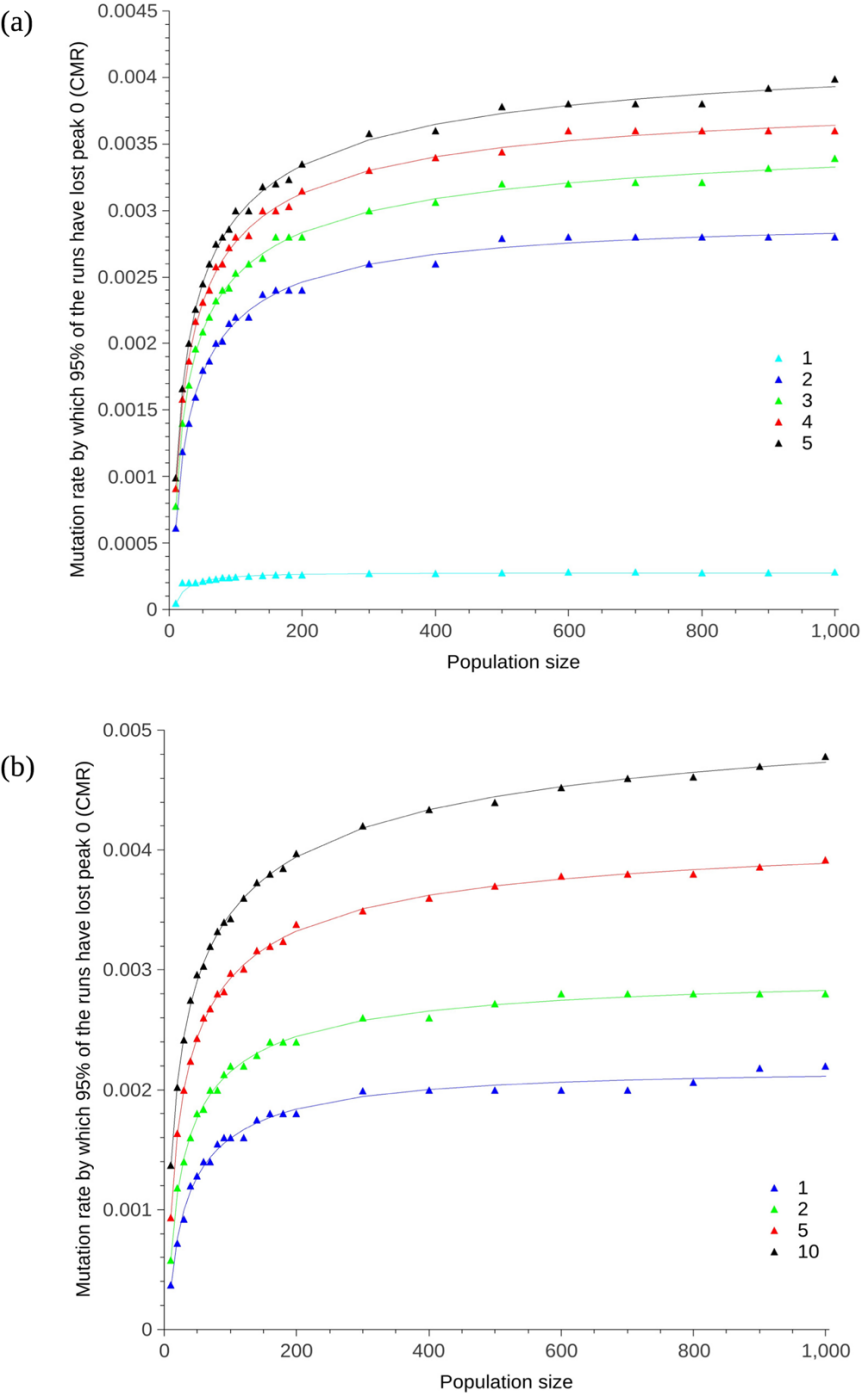
(**a**) CMR plotted for varying number of crossovers. The number of crossover events per reproduction is given in the legend. The exponential lines were obtained by curve-fitting using R with a least squares method. Error bars are not plotted, but are small, for instance $3.91\times {10}^{-03}$ ($3.866\times {10}^{-03}$–$3.954\times {10}^{-03}$, 95% confidence interval) and $2.75\times {10}^{-04}$ ($2.740\times {10}^{-04}$–$2.760\times {10}^{-04}$, 95% confidence interval) for uppermost (5 crossovers) and lowest (1 crossover) points (population size 900) respectively. (**b**) CMR plotted for varying number of chromosomes. The number of chromosomes each gene was split into is given in the legend. The exponential lines were obtained by curve-fitting using R with a least squares method. Error bars are not plotted, but are small, for instance $4.73\times {10}^{-03}$ ($4.703\times {10}^{-03}$–$4.757\times {10}^{-03}$, 95% confidence interval) and $2.75\times {10}^{-04}$ ($2.740\times {10}^{-04}$–$2.760\times {10}^{-04}$, 95% confidence interval) for uppermost (10 chromosomes) and lowest (1 chromosome) points (population size 900) respectively. Population size was varied from 10 up to 1,000 and each individual consisted of 1 gene of 1,000 bp in length. Peak 0 had a radius of 2 and peak 1 a radius of 5. The Hamming distance between the peaks was 10. The number of crossovers per reproduction was increased from 1 (as per previous experiments) to 5, as per the legend in (**a**). The number of chromosomes per gene (given in the legend) was increased from 1 (as per previous experiments) to 10, as per the legend in (**b**).

The previous results, and those of Aston *et al*.^9^, used a simulation model in which every sequence underwent a single crossover per reproduction. Increasing the number of crossovers per reproduction from 1 up to 5 lead to an increase in CMR, with the largest increase occurring when crossover was increased from 1 per reproduction to 2. (Fig. 4(a)). Each gene was also split into up to 10 chromosomes, with 1 recombination event per chromosome. Increasing the number of chromosomes from 1 up to 10 lead to an increase in CMR within the same order of magnitude (Fig. 4(b)). The exponential relationship between CMR and population size was maintained for any number of crossovers or chromosomes. Two-way ANOVA indicates that the magnitude of the CMR will increase with either the number of crossover events (p < 0.0001, *α* = 0.05, F(4,88) = 342.30), or the number of chromosomes (p < 0.0001, *α* = 0.05, F(3,66) = 464.10) for the null hypothesis that there will be no increase in CMR with either the number of crossover events or chromosomes respectively (see Supplementary Tables 3 and 4).

### Increasing the number of genes produces CMRs similar to biological mutation rates

**Figure 5:**
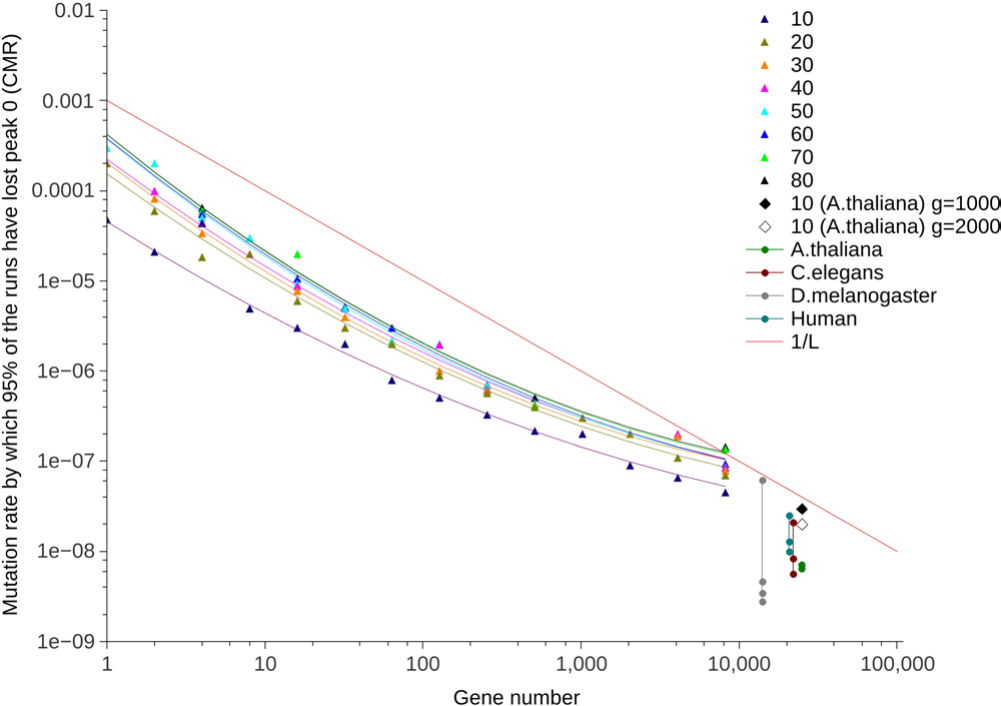
CMR plotted alongside gene number for varying population sizes. Data are shown for population sizes 10 to 80 with results plotted on a log log scale. Peak 0 had a radius of 2 and peak 1 a radius of 5. The Hamming distance between the peaks was 10. Gene length was kept constant at 1,000, while gene number was doubled from 1 up to 8,192. The corresponding quadratic lines were obtained by curve-fitting using R. Error bars are not plotted, but are small, for instance $5.26\times {10}^{-08}$ ($4.100\times {10}^{-08}$–$6.400\times {10}^{-08}$, 95% confidence interval) and $1.25\times {10}^{-07}$ ($1.000\times {10}^{-07}$–$1.500\times {10}^{-07}$, 95% confidence interval) for uppermost (population size 80) and lowest (population size 10) points (gene number 8192) respectively. A line representing 1/L, where L is gene length, is plotted for reference. Population sizes shown represent the steep part of the curve in Fig. 2 before it levels out. Population size 10 was also run with 25,000 genes, the correct range for the plant *A. thaliana*. Gene length was set to 1,000 bp to match the other runs or 2,000 bp to bring it closer to *A. thaliana*’s gene length. For reference, the range of per base mutation rates from Table 1 is shown for *A. thaliana*, *Caenorhabditis elegans* (nematode worm), *Drosophila melanogaster* (fruit fly), and humans (with gene number estimates from^24,64,65^ and^27^ respectively).

As increasing sequence length has been seen to decrease CMR (Fig. 2), increasing the number of genes was also expected to decrease CMR. As per previous experiments, the simulation model was run with a minimal yet biologically realistic gene length of 1,000. However, the number of genes was then doubled from *n* = 1 up to *n* = 8,192. The CMR was recorded as the mutation rate at which *any* of the possible *n* genes was lost in 95% of runs within 10,000 generations. Figure 5 shows the CMR decreases by up to three orders of magnitude as gene number increases from 1 to 8,192, bringing the CMR to within an order of magnitude of the biological mutation rates listed in Table 1. Curve-fitting using R showed the results follow quadratic curves; these can be seen to become closer as population size is increased, indicating the decrease in the rate of change of CMR with increasing population size seen previously (e.g., Fig. 2). Two-way ANOVA indicates that increasing the number of genes in the simulation (while keeping gene length constant) will lower the CMR (p < 0.0001, *α* = 0.05, F(13, 91) = 31.62 for the null hypothesis that there will be no decrease in CMR with an increase in the number of genes) (see Supplementary Table 5).

Population size 10 was also run with 25,000 genes of length 1,000 or 2,000 bp to bring the gene number to within the correct range for the plant *A. thaliana* ^24,25^. Increasing the gene number decreased the CMR further to within an order of magnitude of the per base per generation mutation rate for *A. thaliana* which is given as 7.1 × 10^−9^ (Table 1). Figure 5 shows per base mutation rate estimates for *A. thaliana*, *C. elegans* (nematode worm), *D. melanogaster* (fruit fly), and humans taken from Table 1, each of which are within an order of magnitude of the simulation results for 25,000 genes for population size 10. It should be noted that results relating to the relationship between population size and the number of genes (shown in Fig. 5) were presented at the Artificial Life 2016 conference^23^.

## Discussion

We previously showed that population size influences the CMR that populations can tolerate before fitter individuals are outcompeted by those that have a greater mutational robustness in artificial haploid and diploid populations with a small number of individuals^9^, a result which we demonstrate here to have relevance beyond artificial systems. The mutation rates in Table 1 are up to 10 orders of magnitude lower than the CMR reported in Aston *et al*.^9^. Increasing the sequence length lowered the CMR while maintaining the exponential dependence on population size (Fig. 2); increasing the sequence length by a factor of 10 decreases the CMR by a factor of 10. The relationship between sequence length and CMR is comparable to the relationship between sequence length and error threshold already reported^16–18^; consistency with the existing results for error threshold increases confidence in our novel results for the CMR. The Hamming distance between the two peaks determines the size of the neutral space between them in which individuals can mutate without loss or gain in fitness. Conversely, increasing the radius of the peaks decreases the neutral space. Scaling up both the distance and radius by parameter S lead to an increase in the CMR (Fig. 3). When S = 1, the peaks and space between them take up 2.4% of the genome. When S = 2, this doubles so that the peaks and space between them take up 4.8%. When S = 1, the peaks alone take up 1.4% of the genome versus 2.8% when S = 2; the neutral space decreases by 1.4% when the peak radii are doubled from 1 to 2. Decrease in neutral space means there is more chance of a mutation being non-neutral; mutation rate is expected to be minimised to a greater extent when there is a greater chance of a mutation affecting fitness. Increase in S also means an increase in the mutational robustness of peak 1. CMR increases with S therefore the increase in robustness of peak 1 to mutation exceeds the effect of the increase in non-neutral space. In addition to mutation, individuals move around the landscape by way of crossover. In biological species, the number of crossovers per chromosome and per meiosis is tightly controlled^26^. At mutation rates close to the CMR, recombination acts to pull the few remaining individuals off peak 0 towards the rest of the population on peak 1, leading to survival-of-the-flattest at a lower mutation rate than if recombination was not present. Increasing the number of crossovers from 1 to 5 increased the CMR at population size 1,000 from 0.00028 to 0.0037, with an increase in CMR of one order of magnitude when crossover was increased from 1 to 2 (Fig. 4(a)). Increasing the number of chromosomes affected the number of crossover events per reproduction and therefore also lead to an increase in CMR (Fig. 4(b)).

While it is clear that increasing the sequence length decreases the CMR more so than varying the mutational robustness (peak radii), the number of bases different between alleles (distance between the peaks), or the number of recombination events (both through directly increasing the number of crossovers per reproduction, or indirectly by increasing the number of chromosomes), the CMR remained between two and four orders of magnitude higher than existing estimates of wild-type biological mutation rate (Table 1). Increasing the number of genes to within a biologically-relevant range was expected to lead to CMRs close to the range of wild-type mutation rates in Table 1. Consistent with this, when gene length is kept constant, doubling the number of genes leads to a reduction in the CMR at which 95% of runs lose peak 0 for at least one gene (Fig. 5). The magnitude of this reduction is variable, but occurs across all population sizes shown in Fig. 5. In biological organisms, there is a lower limit on mutation rate as defined by the drift-barrier hypothesis^19^; it is expected biological mutation rates will exist somewhere between this lower limit and the CMR. Figure 5 shows a drop in CMR in the order of three magnitudes as gene number increases from 1 to 8,192. This brings the CMR to within an order of magnitude of the estimates of wild-type biological mutation rates listed in Table 1. For example, population size 10 was run with 25,000 genes of length 1,000 or 2,000 bp to bring the gene number and length to within the correct range for *A. thaliana*. This decreased the CMR further to within an order of magnitude of the per base per generation mutation rate for *A. thaliana* given in Table 1. Figure 5 also shows per base mutation rate estimates for *C. elegans* (nematode worm), *D. melanogaster* (fruit fly), and humans taken from Table 1, all of which are also within an order of magnitude of the simulation results for 25,000 genes. The mutation rates for *A. thaliana* and *C. elegans* are at or below the predicted CMR while *D. melanogaster* is slightly higher but likely to be below the predicted CMR for a population size greater than 10 based on the trend in Fig. 5. It is notable that the genome size estimates for multicellular eukaryotes used in Fig. 5 are based on numbers of protein coding genes. Protein coding sequences account for a relatively small proportion of the total genome length in such organisms (1.2% in humans^27^), but much more of the sequence is functional at some level, probably at least 9%^28^, with estimates of up to 80% in humans^27,29^, (albeit this last figure is likely to be a substantial over-estimate^30^). This means that the genome size at which these biological mutation rates are plotted in Fig. 5 is a minimal estimate, the true value being substantially, perhaps an order of magnitude, higher, therefore putting their observed mutation rates closer to the CMRs estimated by simulation. This is an important contribution; it is the first time the simulation model has been assigned input and produced output within range for a given biological organism.

Bringing the CMR into the biological range is an important step in the development of an *in silico* simulation to directly model the evolution of biological species existing in small populations. Future work will build on this, with the potential to further study the effect on CMR using parameter values for the model organism *A. thaliana*, for example, incorporating its five chromosomes into the simulation model. There is also potential to both develop and test the applicability of the model to a more diverse range of biological species. Specifically, the simulation model has been developed to begin to incorporate features to allow it to be applied to prokaryotic as well as eukaryotic species through the introduction of lateral gene transfer^31^; the transfer of genetic material via lateral gene transfer or transposable elements will further influence the population’s movement in sequence space. There is also potential to use a developed version of the simulation model to simulate evolution of bacteria in an environment in which there is antibiotic present; resistance alleles may be represented by the two-peaks where each peak is modelled on known evolution of antibiotic resistance. In conjunction with wet lab experiments, this has the potential to enable the model to simulate and ascertain *in silico* the efficacy of combined antibiotic administration on the evolution of antibiotic resistance. In eukaryotes, prediction of the CMR for populations of varying sizes will enable identification of the optimum mutation rate, a crucial parameter in the evolution of small populations where CMR is known to vary significantly; this has the potential to influence understanding of populations undergoing a bottleneck, under stress, and subsequent conservation strategy for populations on the brink of extinction.

## Methods

### Model development

The models defined in Aston *et al*.^9^ used arbitrary values for parameters selected for their suitability to provide results within a small time frame. Derelle *et al*.^32^, Sharma *et al*.^33^, and Lewin^34^ list the length of genes for various biological organisms at between approximately 1,000 to 140,000 bp; the sequence length of 30 used to produce the results in Aston *et al*.^9^ is small when compared with the length of genes found in a wide range of species. Unless stated otherwise, 1,000 bp was selected to be a suitable sequence length for study as it is biologically realistic yet small enough to minimise runtime. If each peak in the two-peak landscape (Fig. 1) is considered to represent a set of alleles (variants of a gene), i.e., peak 0 is one set of alleles, peak 1 is another set of alleles of the same gene, estimates of genetic distance between alleles for various genes can be seen to be analogous to the distance between the peaks. Distance between the peaks can therefore be considered to fall within the range of 1 and 56 polymorphisms (Table 2). Similarly, the number of polymorphisms was estimated to be at most 13 (including non-coding regions) within human genes studied by Cargill *et al*.^35^, a subset of which are given in Table 2. In terms of the simulation model, there is no part of the fitness landscape in which individuals cannot be chosen to reproduce. In a model which allowed individuals that lose the peaks to ‘die’, non-lethal neutral parts of the landscape would be represented by plateaus on the peaks; in the simulation model the neutral space is considered to be any part of the landscape apart from the peaks. To test the effect of scaling both the distance between peaks and the radius of the peaks by a given parameter S, peak 0 and peak 1 were given an initial radius of 2 and 5 respectively, while the distance between the top of the peaks was set to 10. S was set to equal 1 up to 10 and multiplied by the radii and distance. The resulting distances covered the range defined by Table 2. To test the effect of recombination, the number of crossovers per reproduction was increased from 1 up to 5. This was kept constant for the duration of each set of runs; there is variation in the rate of crossover within biological species (for example, Table 3 (Table 1 in^26^), but to ascertain the effect of varying the number of crossovers per reproduction it was necessary to keep the number constant. Allowing variation within runs would have also increased the number of required run repetitions in order to counteract the increase in noise. The genome was split into up to 10 chromosomes, with 1 recombination event per chromosome. To test the effect of increasing gene number, the gene length was kept at 1,000 bp, but gene number was increased from 1, doubling each time up to 8,192. Gene number experiments were done for population sizes of 10 up to 80, i.e., those representing the exponential phase of the data in Fig. 2 before it plateaus. This subset of population sizes was chosen to minimise runtime.

**Table 3:** Crossover rates in *A. thaliana* taken from Table 1 in Giraut *et al*.^26^.

	Female	Male	Ratio Male/Female
Number of COs analysed	5003	8532	
Size genetic map (cM)	332	575	
COs per cell	6.65	11.15	1.67
COs per chromosome 1 bivalent	1.63	2.85	1.75
COs per chromosome 1 bivalent corrected*	1.63	3.18	1.95
COs per chromosome 2 bivalent	1.19	1.89	1.58
COs per chromosome 3 bivalent	1.29	2.14	1.66
COs per chromosome 4 bivalent	1.10	1.71	1.56
COs per chromosome 5 bivalent	1.44	2.58	1.79

*Values given for male bivalent with and without correction for segregation bias (see Giraut *et al*.^26^).

Based on the information in Table 1 and the value of 2,232 bp mean gene size (minus introns) given by Derelle *et al*.^32^, *A. thaliana* (thale cress) was selected as a target model organism; it has a relatively short gene length which makes it an ideal candidate for *in silico* simulation with minimised runtime. It is a plant native to Eurasia, with an effective population size of between 250,000 to 300,000 ^36^, known to contain 25,498 genes encoding proteins from 11,000 families^24^. More current estimates of gene number are slightly higher but still within a close range for the purpose of the model^25^. *A. thaliana* is a hermaphroditic plant in which male and female crossover occurs within the same plant cells^26^. Chromosomes, of which there are five, recombine 1.7 times more in male meiosis when compared with female meiosis, with male crossover rates remaining very high at both ends of each chromosome while female rates are very low. Analysed crossover rates in *A. thaliana* are as listed in Table 3 which was taken from Table 1 in Giraut *et al*.^26^. The simulation was run for population size 10 with a gene length of 1,000 bp (as per the previous runs) or 2,000 bp (range of *A. thaliana*), but with 25,000 genes to bring the gene number into the range of *A. thaliana*. Population size 10 was chosen to minimise runtime.

### Simulation model

The simulation model used a two-peak fitness landscape (Fig. 1), with the height of peak 0 constant at 15 and the radius 2, the height of peak 1 constant at 10 and the radius 5, and the Hamming distance between the peaks set at 10 as per Aston *et al*.^9^ (unless stated otherwise). Fitness was defined as a relative score therefore the choice of peak height was not important; peak 0 was set to have a maximum fitness score greater than that of peak 1. Each individual consisted of one randomly assigned maternal and one paternal sequence of alphabet size 4, and each sequence was split into *n* genes of length *L*. Each gene had an associated target sequence of length *L* corresponding to peak 0 and a target sequence corresponding to peak 1. For example, if *n* is set to 4, there will be target sequences corresponding to peaks 0_1_1_1_, 0_2_1_2_, 0_3_1_3_, and 0_4_1_4_. For simplicity, each peak 0 was set to be all 0 s and each peak 1 was randomly generated to be Hamming distance 10 away. The simulation was initialised so that half of the population was on the top of peak 0 and half on the top of peak 1. Recombination was limited to one event per replication unless otherwise stated.

Mutation was done according to a given probability of per base mutation. A random number K was generated from a binomial distribution of L trials (where L is the length of the sequence) with M probability of mutation. K positions in the sequence were then sampled and mutated to a different base. There was an equal chance of mutation to any of the other three possible values. For each individual, the fitness of each of its *n* genes was calculated as the Hamming distance of the maternal and paternal sequences relative to each peak. The maternal fitness value relative to peak 0 was compared with the maternal fitness value relative to peak 1 and the highest of these selected to give a single maternal fitness value. This was repeated for the paternal sequence. The resulting maternal and paternal fitnesses were compared and subsequently designated as ${f}_{{\rm{\max }}}$ and ${f}_{{\rm{\min }}}$. The final relative fitness of each gene was calculated as $f=(\lambda \times {f}_{{\rm{\max }}})+(\mathrm{(1}-\lambda )\times {f}_{{\rm{\min }}})$, where *λ* is the dominance parameter which determines the relative contribution of the maternal and paternal sequences to the overall fitness of the individual. Rather than select one to be completely dominant over the other (i.e., *λ* = 1.0), *λ* was set to equal a fraction below 1.0 (0.999999999999999 specifically). If *λ* = 1.0, the fitness of only one allele is taken into account, while the other can be anywhere in the fitness landscape drifting neutrally; setting *λ* to just below 1.0 ensures both the maternal and paternal alleles contribute to the fitness of the individual. When each individual had more than one gene, the overall fitness of the individual was taken to equal the minimum fitness out of the *n* genes present. In biological species, there is complex interaction between genes (epistasis)^37^. The minimum fitness was selected as the overall individual fitness as a realistic form of epistasis that avoids the situation in which one gene on top of the highest peak could mask the loss of the peaks by multiple other genes in the simulation. It is also representative of the fact that, in biological species, a large proportion of genes is classed as essential; that is, gene deletion results in lethality or infertility in a particular environment^38–41^. During the replication step, three individuals were selected at random, with two being designated parents and one to be replaced with the resulting child. The individual to be replaced was determined based on the fitnesses of the three individuals: there was an equally small chance of either of the two fittest of the three being replaced (25%), and a 50% chance of replacing the least fit. This ratio ensures that there is potential for any individual to be chosen for replacement, allowing loss of the fittest peak. This step was repeated until each individual in the population had been chosen exactly once to undergo reproduction (or there were less than three remaining to select); this represents one discrete generation. Such non-overlapping generations exist in nature, and that discreteness matters for population dynamics^42^. For example, periodic insects, such as 13-year cicadas, have non-overlapping generations^43^ as do some salmon and many annual plants, both wild and agricultural. Non-overlapping generations are also widely used laboratory tests of biological evolution, using model species such as *C. elegans* (e.g.^44^,) and *D. melanogaster* (e.g.^45^). Our simulation was run for a range of population sizes to confirm the curves observed in previous experiments^8,9^, was observed as the length and number of genes was increased.

To allow the simulation to complete within a realistic time frame, it was optimised to cease running when any one gene had lost peak 0; this was all the information required to determine the CMR, which was recorded as the mutation rate at which 95% of 2,000 runs lost peak 0 within 10,000 generations for any of the possible *n* genes. This means that a population of individuals with a single gene and a population of individuals with 1,000 genes will both cease running when peak 0 is lost for a single gene. The CMR was calculated for each 100 runs to produce 20 CMR values from which the mean was calculated, along with the standard deviation and the 95% confidence interval. Each set of expected CMR values for each variable being tested was analysed by two-way ANOVA, with main effects of population size and the variable of interest (e.g., sequence length) treated as independent factors. Launching the simulation for various combinations of parameter values was also optimised to allow the mutation rate being tested for a given gene number to progress to the next mutation rate once 100 out of the possible 2,000 runs (corresponding to 5%) have kept peak 0 for the duration of the simulation. Once this threshold has been exceeded, less than 95% of the 2,000 runs will have lost peak 0, and the CMR will not have been reached. Runs started with a very low per base mutation rate ($1\times {10}^{-8}$). The first digit was then incremented up to the next order of magnitude (i.e., $2\times {10}^{-8}$, $3\times {10}^{-8}$, etc. up to $1\times {10}^{-7}$). This was repeated up to a mutation rate of $1\times {10}^{-2}$. This enabled identification of the order of magnitude of the CMR. Mutation rates within the appropriate order of magnitude for each set of parameter values were then incremented by $0.1\times {10}^{-n}$, i.e., $1.1\times {10}^{-n}$, $1.2\times {10}^{-n}$, $1.3\times {10}^{-n}$, etc. to pinpoint the CMR. While this helped significantly with runtime, further optimisation will be required in the future; it is currently not feasible to run the simulation for a wide range of population sizes and mutation rates for gene numbers at the upper end of the biological range.

### Data Availability

Datasets generated and analysed are openly available at https://doi.org/10.5281/zenodo.837869. The source code for the simulation model is openly available at http://doi.org/10.5281/zenodo.581389.

## Electronic supplementary material

Supplementary Information
