# Supplementary material for: Critical Mutation Rate has an Exponential Dependence on Population Size for Eukaryotic-length Genomes with Crossover
Source: Sci Rep. 2017 Nov 14;7:15519. doi: 10.1038/s41598-017-14628-x (PMC5686101; doi:10.1038/s41598-017-14628-x)
Supplement: Supplementary file 1 — Supplementary Information [file 41598_2017_14628_MOESM1_ESM.pdf]

Supplementary Information

Title: Critical Mutation Rate has an Exponential Dependence on Population Size for Eukaryotic-length Genomes with Crossover

Authors: Elizabeth Aston, Alastair Channon, Roman V. Belavkin, Danna R. Gifford, Rok Krašovec, and Christopher G. Knight.

Supplementary Table 1: Two-way ANOVA for varying sequence lengths (bp) and population sizes.

Source of Variation	*SS*	*df*	*MS*	*F*	*P-value*
Sequence length	0.0015	8	0.0002	453.0879	3.9861×10^{-113}
Population size	3.2044×10^{-5}	22	1.4566	3.5355	1.398×10^{-6}
Error	7.2508×10^{-5}	176	4.1200×10^{-7}		
Total	0.0016	206			

Supplementary Table 2: Two-way ANOVA for varying values of scale parameter S and population sizes.

Source of Variation	*SS*	*df*	*MS*	*F*	*P-value*
S	2.9072×10^{-6}	9	3.2300×10^{-7}	89.0702	1.0164×10^{-64}
Population size	5.0337×10^{-6}	22	2.2881×10^{-7}	63.0902	3.9548×10^{-77}
Error	7.1807×10^{-7}	198	3.6266×10^{-9}		
Total	8.6590×10^{-6}	229			

Supplementary Table 3: Two-way ANOVA for varying number of crossover events and population size.

Source of Variation	*SS*	*df*	*MS*	*F*	*P-value*
Crossover events	1.1610×10^{-4}	4	2.9024×10^{-5}	342.2993	9.7547×10^{-53}
Population size	3.4162×10^{-5}	22	1.5528×10^{-6}	18.3131	4.9715×10^{-24}
Error	7.4617×10^{-6}	88	8.4792×10^{-8}		
Total	1.5772×10^{-4}	114			

Supplementary Table 4: Two-way ANOVA for varying number of chromosomes and population size.

Source of Variation	*SS*	*df*	*MS*	*F*	*P-value*
Chromosomes	5.3151×10^{-5}	3	1.7717×10^{-5}	464.0915	2.7867×10^{-44}
Population size	4.0918×10^{-5}	22	1.8599×10^{-6}	48.7199	1.6804×10^{-32}
Error	2.5196×10^{-6}	66	3.8176×10^{-8}		
Total	9.6588×10^{-5}	91			

Supplementary Table 5: Two-way ANOVA for varying number of genes and population size.

Source of Variation	*SS*	*df*	*MS*	*F*	*P-value*
Number of genes	6.0455×10^{-7}	13	4.6504×10^{-8}	31.6218	4.0578×10^{-28}
Population size	2.5319×10^{-8}	7	3.6169×10^{-9}	2.4595	0.0234
Error	1.3383×10^{-7}	91	1.4706×10^{-9}		
Total	7.6369×10^{-7}	111			
